# Implementing an automated monitoring process in a digital, longitudinal observational cohort study

**DOI:** 10.1186/s13075-021-02563-2

**Published:** 2021-07-07

**Authors:** Lisa Lindner, Anja Weiß, Andreas Reich, Siegfried Kindler, Frank Behrens, Jürgen Braun, Joachim Listing, Georg Schett, Joachim Sieper, Anja Strangfeld, Anne C. Regierer

**Affiliations:** 1 Epidemiology Unit, German Rheumatism Research Centre (DRFZ), Charitéplatz 1, 10117 Berlin, Germany; 2 Rheumatology, Goethe University, Frankfurt, Germany; 3 Rheumazentrum Ruhrgebiet, Herne, Germany; 4 Rheumatology and Immunology, Universitätsklinikum Erlangen, Erlangen, Germany; 5 Charité — Universitätsmedizin Berlin, CBF, Berlin, Germany

**Keywords:** Data validation, Observational study, Data monitoring, Spondyloarthritis

## Abstract

**Background:**

Clinical data collection requires correct and complete data sets in order to perform correct statistical analysis and draw valid conclusions. While in randomized clinical trials much effort concentrates on data monitoring, this is rarely the case in observational studies- due to high numbers of cases and often-restricted resources. We have developed a valid and cost-effective monitoring tool, which can substantially contribute to an increased data quality in observational research.

**Methods:**

An automated digital monitoring system for cohort studies developed by the German Rheumatism Research Centre (DRFZ) was tested within the disease register RABBIT-SpA, a longitudinal observational study including patients with axial spondyloarthritis and psoriatic arthritis. Physicians and patients complete electronic case report forms (eCRF) twice a year for up to 10 years. Automatic plausibility checks were implemented to verify all data after entry into the eCRF. To identify conflicts that cannot be found by this approach, all possible conflicts were compiled into a catalog. This “conflict catalog” was used to create queries, which are displayed as part of the eCRF. The proportion of queried eCRFs and responses were analyzed by descriptive methods. For the analysis of responses, the type of conflict was assigned to either a single conflict only (affecting individual items) or a conflict that required the entire eCRF to be queried.

**Results:**

Data from 1883 patients was analyzed. A total of n = 3145 eCRFs submitted between baseline (T0) and T3 (12 months) had conflicts (40–64%). Fifty-six to 100% of the queries regarding eCRFs that were completely missing were answered. A mean of 1.4 to 2.4 single conflicts occurred per eCRF, of which 59–69% were answered. The most common missing values were CRP, ESR, Schober’s test, data on systemic glucocorticoid therapy, and presence of enthesitis.

**Conclusion:**

Providing high data quality in large observational cohort studies is a major challenge, which requires careful monitoring. An automated monitoring process was successfully implemented and well accepted by the study centers. Two thirds of the queries were answered with new data. While conventional manual monitoring is resource-intensive and may itself create new sources of errors, automated processes are a convenient way to augment data quality.

**Supplementary Information:**

The online version contains supplementary material available at 10.1186/s13075-021-02563-2.

## Background

Rabbit-SpA (*R*heumatoid *a*rthritis: o*b*servation of *bi*ologic *t*herapy—*sp*ondylo*a*rthritis) is an observational longitudinal cohort study, initiated by the German Rheumatism Research Center (DRFZ) in 2017 [1]. It aims to describe the long-term safety and effectiveness of treatment with biologic and targeted synthetic disease-modifying antirheumatic drugs (b/tsDMARDs). Patients with a diagnosis of axial spondyloarthritis (axSpA) or psoriatic arthritis (PsA) are enrolled at the beginning of either a new therapy with a b/tsDMARD or a conventional systemic therapy after the failure of at least one previous systemic therapy. While the German biologics register RABBIT, which has been enrolling patients with rheumatoid arthritis since 2001, is still paper-based [2], RABBIT-SpA, as well as other international rheumatological registries, records disease data electronically via a digital documentation system [3–8].

Unlike randomized clinical trials (RCTs), data validation for critical variables is rarely reported in observational studies. Although many data monitoring and auditing methods in RCTs have been frequently reported and compared [9–11], on-site source data verification (SDV) is still a common procedure to ensure high data quality in RCTs [12]. Since SDV is a very expensive and personnel-intensive method [13] and observational studies are often limited in its financial, time, and personnel resources, this approach is not feasible in most cases. Unlike RCTs, data are not collected exclusively for the study, so it is much more difficult to obtain completeness of the data requested on the CRF as only data from routine clinical care is available. A further challenge in many longitudinal observational studies lies in the fact that there is no fixed recruitment period and new patients are constantly being recruited into the study. This makes it impossible to perform data cleaning for each time point at once, sending out lists of queries at a few time points only. Rather, like the recruitment, the monitoring must be an ongoing process.

One of the advantages of observational studies over clinical studies lies in the evaluation of long observation periods [14]. For this purpose, it is essential that the data quality remains high over time and that the amount of missing or implausible data is minimized as much as possible. For cohort studies that run for several years, it is important not to strain the motivated and committed study participation of all study participants by requesting queries too late. This poses particular challenges for the development of data validation measures.

Monitoring a large amount of data for plausibility, especially in long-term cohort studies, is a very demanding and time-consuming task. If every data record must be checked manually, this is also very personnel-intensive [14]. Given the digital nature of the project, our aim was to overcome the manually driven data validation process to save resources, accelerate the process, and improve data quality. We aimed to develop a data validation system that allows a timely querying of data and is as convenient as possible for the participating sites as well as for the study leadership (DRFZ) but is almost as thorough as in a randomized clinical trial to enhance data quality. The objective of this manuscript is to describe and analyze the digital data validation process within the observational study Rabbit-SpA.

## Methods

### RABBIT-SpA-register description

Patients and physicians complete pseudonymized electronic case report forms (eCRF) in a web-based documentation system, which was specifically designed for RABBIT-SpA, without the need to install specific desktop software. Physicians and patients complete questionnaires after 3 and 6 months and thereafter twice a year for 5 years with the possibility of extension to up to 10 years. Sociodemographic parameters, laboratory values, clinical parameters, treatment details, and physician and patient endpoints as well as safety events are collected. The DRFZ receives physician and patient questionnaires electronically. Configurable roles with individual rights are assigned to each participant (physician, study nurse, patient, technical administration, and trustee and research group). This ensures that only the content and data assigned to the role can be accessed at any time, thus complying with the applicable German and European data protection regulations. To allow the study centers and patients a convenient entry into the system, several instructional videos, for example on how to add patients and how to access the eCRFs, were created and posted on the study homepage. As the documentation is often carried out during consultation hours with limited time, the eCRFs and the documentation system have to be as intuitive and user friendly as possible. To achieve this, several functions have been implemented. The system reminds the participants of pending questionnaires. If a questionnaire has not been completed in a predefined period, it automatically expires and will be submitted automatically. In case the required data such as laboratory values are not yet completely available, the eCRF can still be submitted. Therefore, the eCRF can be submitted despite implausible or missing values. Only the date of the survey and the information on medication are mandatory fields, without which the eCRF cannot be submitted manually. Although plausibility checks have also been implemented on patient eCRFs, the monitoring process refers exclusively to eCRFs filled in by physicians.

### Plausibility checks on the eCRF

The values entered on the eCRF are validated directly upon entry. If an implausible value is entered, for example an out of range value, an in-page alert immediately occurs to indicate that a plausible value needs to be entered. Before submitting the eCRF, the whole document is checked for missing or implausible data. Some values can be validated directly during entry; other answers must be compared to related answer values. When submitting an eCRF, each field is checked and validated. If the mandatory fields (inclusion date, year of birth, gender, and treatment details) are filled out as defined, the eCRF can still be submitted despite other missing values. Therefore, it remains possible to enter implausible or contradictory data despite immediate automatic data validation on the eCRF. The submitted eCRFs form one data set, which is the basis for rechecking thoroughly via the automated monitoring system.

### Conflict catalog

To specify possible conflicts, the eCRFs were examined systematically. All potential errors were compiled in a table, called “conflict catalog”, with which the data set is checked. The number of possible conflicts varies for different eCRFs depending on diagnosis and visit. A conflict can either be an eCRF that is completely missing (“entire eCRF-conflict”) or affect single items in the eCRF (“single conflict”). These single conflicts were categorized into the types missing values, range, and date conflicts. See supplement for more details (page 6).

The conflict catalog contains all information that is needed to create queries. It is used in SAS and in the for RABBIT-SpA and created the monitoring database. The conflict label contains the text of the request to the physician that is displayed along with necessary additional variables. In order to facilitate answering these queries, the part of the eCRF is displayed in which the conflicts were found.

Additional variables (“unknown” or “not done” or the “value is correct”) are answering options with which the physician has the possibility to answer the query without changing the original value.

### The monitoring database

The monitoring database was set up to store the data and document changes. It further controls the process of query generation and management. It groups the data from the conflict list and creates the queries. A query summarizes the occurring conflicts per patient and time point of visit and can contain one or more conflicts. Queries and the corresponding answers are saved in the monitoring database, making changes in the data traceable. The database saves queries and their response status (answered, submitted but not answered sufficiently, or expired) and corrects the clinical data on basis of query answers given by the physician.

### The monitoring process

**Fig. 1:**
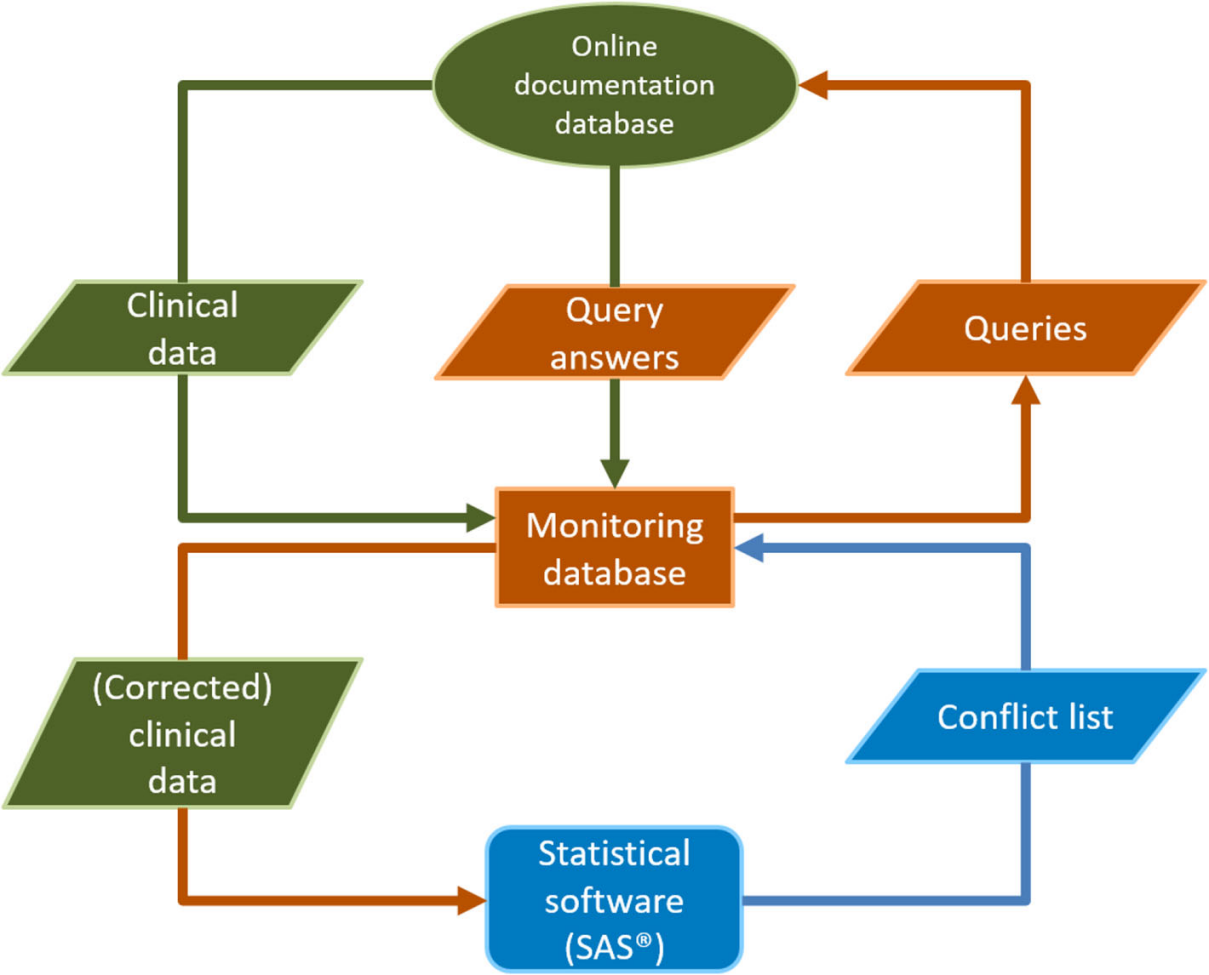
Flowchart of the monitoring process in RABBIT-SpA

Figure 1 gives an overview of the automated monitoring process. The online documentation system provides the clinical data set that is exported by the research group and then processed in the monitoring database. SAS uses the clinical data from the monitoring database and creates a conflict list, which is sent to the monitoring database. The monitoring database summarizes the conflict list to a query list. Once the query list has been created, the monitoring database creates a file to transfer the data to the online documentation system. The information from this file is used by the documentation system to select which parts of the eCRF are displayed to the physician. The queries answered by the physician are downloaded and imported to the monitoring database, which checks the answers for completeness and creates a list of corrections in the clinical data. See supplement for a more detailed description of the process.

### Study population

RABBIT-SpA started in May 2017. For this analysis, all eCRFs of patients whose baseline eCRF was submitted between May 2017 and June 2020 were included.

### Analysis

Descriptive methods were used to analyze the proportion of queried eCRFs and response status using SAS Enterprise Guide 7.1. The mean value of the single conflicts results from the number of single conflicts per query containing only single conflicts. The calculation of the response options (answered and unanswered) is based on the number of single conflicts queried.

## Results

**Fig. 2:**
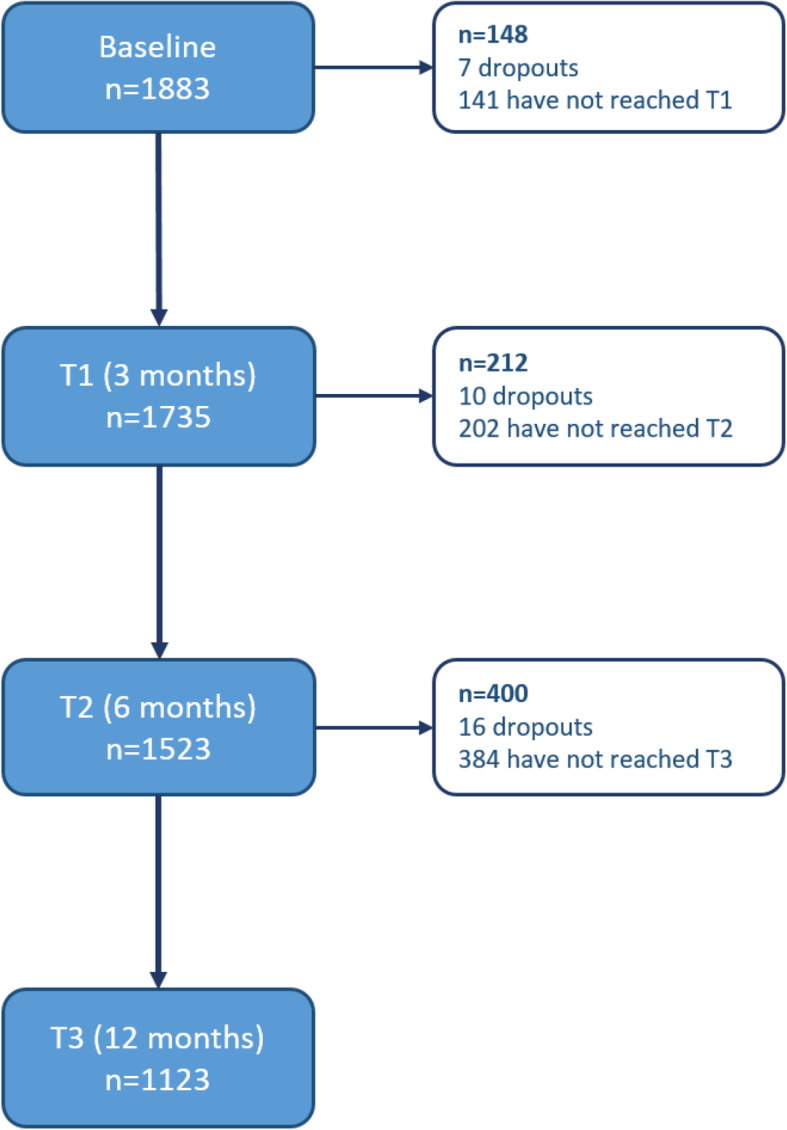
Flowchart of included patients

A total of 1883 submitted baseline eCRFs from 986 patients with axSpA and 897 patients with PsA were included (Table 1). A flowchart of included patients per visit is shown in Fig. 2.

**Table 1:** Baseline characteristics in RABBIT-SpA

	axSpA	PsA	Total
N	986	897	1883
Age, mean (SD)	44.3 (13)	51.6 (12)	47.8 (13)
Female, n (%)	436 (44)	530 (59)	966 (51)
Duration of symptoms (years), mean (SD)	12 (11)	9.4 (9)	10.8 (10)
Disease duration (years), mean (SD)	6.9 (9)	6.5 (8)	6.7 (8)
CRP ≥ 5 mg/L, n (%)	446 (56)	185 (44)	631 (52)
BMI ≥ 30, n (%)	244 (25)	330 (37)	574 (31)
Disease activity, mean (SD)	5.5 (2)	5.2 (2)	5.4 (2)
Patient global, mean (SD)	5.8 (2)	5.7 (2)	5.7 (2)
Patient pain, mean (SD)	5.7 (2)	5.5 (2)	5.6 (2)

*SD* standard deviation, *CRP* C-reactive protein, *BMI* body mass index

At baseline (T0), 1198 of 1883 submitted eCRFs (64%) had conflicts (Table 2). After 3 months (T1) and 6 months (T2), there were 44% and 40% of eCRFs, for which conflicts were queried. Slightly more than half of all eCRFs had conflicts after 12 months (T3) (51%).

**Table 2:** Response status of all requested conflicts from T0 to T3

	T0 (baseline)	T1 (after 3 months)	T2 (after 6 months)	T3 (after 12 months)
No. of submitted eCRFs, N	1883	1735	1523	1123
eCRFs without conflicts, n (%)	685 (36)	976 (56)	908 (60)	550 (49)
eCRFs with conflicts, n (%)	1198 (64)	759 (44)	615 (40)	573 (51)
**Analysis of entire eCRF conflicts**				
No. of entire eCRF conflicts queried, n	12	296	170	133
No. of entire eCRF conflicts answered, n (%)	12 (100)	166 (56)	104 (61)	78 (59)
No. of entire eCRF conflicts unanswered, n (%)	0 (0)	130 (44)	66 (39)	55 (41)
**Analysis of single conflicts**				
No. of eCRFs with single conflicts, n	1186	463	445	440
No. of single conflicts queried, n	2835	732	633	843
Single conflicts per eCRF with single conflicts, mean (SD)	2.4 (3)	1.6 (2)	1.4 (1)	1.9 (2)
No. of single conflict queries answered, n (%)	1949 (69)	430 (59)	409 (65)	585 (69)
No. of single conflict queries unanswered, n (%)	886 (31)	302 (41)	224 (35)	258 (31)

*eCRF* electronic case report form, *SD* standard deviation

Out of all eCRFs with conflicts, at baseline, 1% (n = 12) of eCRFs were queried entirely (Table 2). At T1, T2, and T3, it was 39%, 28%, and 23%, respectively. The proportion of overall query-responses differed between visits. At baseline, all entire eCRF conflicts that were queried were answered (100%). At visit T1 56% and T2 61% of the entire eCRFs that were queried were at least partially filled in. For T3, the percentage of answered entire eCRF conflicts decreased to 59%.

At baseline, 2835 single conflicts were identified in 1198 submitted eCRFs with conflicts (Table 2). This is a mean of 2.4 single conflicts per eCRF. Throughout the follow-up visits, means of 1.4 to 1.9 single conflicts per eCRF were queried. The proportion of overall query-responses hardly differs between visits. About two thirds (59–69%) of the queries were answered with a new value. Thirty-one to 41% remained unanswered.

The most common single conflicts were missing values regarding laboratory results such as CRP (C-reactive protein) and ESR (erythrocyte sedimentation rate). Furthermore, frequently missing parameters were Schober’s test, data on systemic glucocorticoid therapy, and present enthesitis.

## Discussion

This article describes an automated data monitoring system based on the example of the disease register RABBIT-SpA. Monitoring real world data of large observational cohort studies that include several thousand patients is a major challenge. Although it is highly recommended to describe “[...] quality assurance and quality control procedures” [15, 16] when publishing results. We have identified only one publication describing the handling of quality assurance in cohort studies [3]. Data generated by cohort studies support clinical decision-making and guideline recommendations [2, 17–19]. The relevance and acceptance of such studies has increased in the last years and the demands for data quality are coming into focus in a way that was not common before [20, 21].

Monitoring data manually can lead to further errors, for example when editing the conflict list and manually incorporating corrections from the queries. Furthermore, the process of manually driven monitoring systems is a very time-consuming and personnel-intensive task [14].

An automated monitoring system must take into account the challenges and problems that can arise during digital data collection and data cleaning. The prerequisite for automated monitoring is a consistent data structure. This also requires that follow-up eCRFs are consistent in content, design, and data structure.

The benefit of digital data collection, compared to paper-based documentation, is the possibility of giving immediate feedback on missing and implausible data, via plausibility checks directly in the eCRF. The analysis showed that 40–64% of eCRFs, submitted between baseline and T3, had conflicts. Therefore, incorrect or implausible data continued to be received, despite initial error checking on the eCRF. Only 1% of the baseline eCRFs were entire eCRF conflicts. This is because baseline visits are mandatory for study inclusion. However, due to technical reasons, 12 of the questionnaires had only one to three variables that were filled in, which resulted in the entire eCRF being queried as missing. Most of the entire eCRF conflicts were queried at T1 (3 months after baseline). Since RABBIT-SpA is an observational study, patients are invited at the physician’s discretion and some study centers do not regularly perform a patient visit after 3 months, which explains the high number of missing entire eCRFs at this time point. Most of the single conflicts were queried at baseline and T3, which is probably related to the fact that these eCRFs contain more variables, are more complex, and the number of possible conflicts is higher than at the follow-up visits T1 and T2. Whereas 388 conflicts are possible at baseline and 208 at T3, only 118 conflicts are the maximum to be queried at T1 and T2. Thus, the number of eCRFs, that need to be queried, depends on the number of possible conflicts and on the complexity of the eCRF.

The most common single conflicts were missing laboratory values. It is very plausible that in many cases the laboratory results were not yet available at the point of documentation. Therefore, it is of great importance to provide a query system, which allows the completion of these missing values at a later time.

One of the goals of the automated monitoring system was to make the answering of queries as easy as possible for the study centers. Therefore, the queries reflect parts of the eCRF so that they can be easily recognized and answered quickly. In this analysis, 67% of the queries were answered. Positive feedback from participating study centers suggests that a user-friendly monitoring system was successfully created that meets the predefined requirements.

The automated monitoring has proven beneficial on several levels. It is much less personnel-intensive. While one data manager is able to take responsibility for the complete data collection and monitoring of the data on the eCRFs, in the paper-based RABBIT study, it takes six medical documentalists to generate queries [14]. For the participating rheumatology practices, the workload is considerably reduced because the subsequent queries are eliminated and the incorrect data is recognized and reported directly in the system. With an answering rate of 69% of entire eCRF conflicts and 66% of single conflicts, the completeness of data will improve significantly. In a comparison of cohort studies covering pregnancy in rheumatology patients, a significant variation in number of missing values has been described depending on the variable [22]. The extent of missing data in our analysis is considerably lower, compared to other cohorts [22, 23]. Nevertheless, querying the missing values will increase the data quality even further. Whereas the dropout rate is higher in other studies [14, 24], the dropout rate after 1 year of observation is only 1.8% in RABBIT-SpA, which is a remarkable low rate for observational studies. The successful implementation of our monitoring system might be one of the reasons for this low number.

Prior to active monitoring, there was a large amount of incomplete or missing data in our observational cohort study, which is probably typical of this type of study. However, even though almost half of the eCRFs had to be queried, this resulted in only a few (1.1 to 2.4) conflicts per eCRF, which kept the workload for the study centers at an acceptable level. In addition, we can use our monitoring system as a quality management tool as we can identify what percentage of queries are answered, how well they are answered over time, and per participating institution, and thus we can adjust descriptions for queries that are particularly poorly completed.

## Conclusion

Active monitoring can improve the quality and completeness of primary observational data and thus the robustness of results and conclusions. Technical solutions, routines, and processes are available that allow monitoring of large data sets despite limited time and financial resources. Careful development of plausibility checks and rules for queries and the user-friendly presentation to those entering the data are of utmost importance. The successful implementation of a digital automated control could also help to standardize the data collection of collaborative multicenter studies in the future. It is a valuable digital tool to ensure data harmonization while increasing data quality and consistency. Based upon the example presented here, active, automated monitoring of all studies using eCRFs is highly recommended.

## Supplementary Information

**Additional file 1:**  Detailed description of the monitoring process in RABBIT-SpA.

## Data Availability

The data that support the findings of this study are available from German Rheumatism Research Centre but restrictions apply to the availability of these data, which were used under license for the current study, and so are not publicly available. Data are however available from the authors upon reasonable request and with permission of German Rheumatism Research Centre.

## References

[1] Regierer AC, Weiss A, Baraliakos X, Zink A, Listing J, Strangfeld A. RABBIT-SpA: a new disease register for axial spondyloarthritis and psoriatic arthritis. Z Rheumatol. 2019. 10.1007/s00393-019-0613-z 30874933

[2] Meißner Y, Milatz F, Callhoff J, Minden K, Regierer A, Strangfeld A (2020). Register- und Kohortenstudien. Z Rheumatol.

[3] Hetland ML (2010). DANBIO—powerful research database and electronic patient record. Rheumatology..

[4] Watson K, Symmons D, Griffiths I, Silman A (2005). The British Society for Rheumatology Biologics Register. Ann Rheum Dis.

[5] Lapadula G, Ferraccioli G, Ferri C, Punzi L, Trotta F (2011). GISEA: an Italian biological agents registry in rheumatology. Reumatismo..

[6] Canhão H, Faustino A, Martins F, Fonseca JE (2011). Reuma.pt - the rheumatic diseases portuguese register. Acta Reumatol Port.

[7] Pavelka K, Forejtova S, Stolfa J, Chroust K, Buresova L, Mann H (2009). Anti-TNF therapy of ankylosing spondylitis in clinical practice. Results from the Czech national registry ATTRA. Clin Exp Rheumatol.

[8] Uitz E, Fransen J, Langenegger T, Stucki G (2000). Clinical quality management in rheumatoid arthritis: putting theory into practice. Rheumatology..

[9] De S (2011). Hybrid approaches to clinical trial monitoring: practical alternatives to 100% source data verification. Perspectives in clinical research.

[10] Houston L, Probst Y, Martin A (2018). Assessing data quality and the variability of source data verification auditing methods in clinical research settings. J Biomed Inform.

[11] Krishnankutty B, Bellary S, Kumar NB, Moodahadu LS (2012). Data management in clinical research: an overview. Indian J Pharm.

[12] Houston L, Probst Y, Yu P, Martin A (2018). Exploring data quality management within clinical trials. Applied clinical informatics.

[13] Califf RM, Karnash SL, Woodlief LH (1997). Developing systems for cost-effective auditing of clinical trials. Control Clin Trials.

[14] Richter A, Meissner Y, Strangfeld A, Zink A (2016). Primary and secondary patient data in contrast: the use of observational studies like RABBIT. Clin Exp Rheumatol.

[15] Public Policy Committee (2016). Guidelines for good pharmacoepidemiology practice (GPP). Pharmacoepidemiol Drug Saf.

[16] von Elm E, Altman DG, Egger M, Pocock SJ, Gøtzsche PC, Vandenbroucke JP (2007). The Strengthening the Reporting of Observational Studies in Epidemiology (STROBE) statement: guidelines for reporting observational studies. Lancet..

[17] Strangfeld A, Richter A (2015). Wie unterstützen Registerdaten die klinische Entscheidungsfindung?. Z Rheumatol.

[18] Ercole A, Brinck V, George P, Hicks R, Huijben J, Jarrett M, Vassar M, Wilson L, the DAQCORD collaborators (2020). Guidelines for Data Acquisition, Quality and Curation for Observational Research Designs (DAQCORD). J Clin Transl Sci.

[19] Ligthelm RJ, Borzì V, Gumprecht J, Kawamori R, Wenying Y, Valensi P. Importance of observational studies in clinical practice. Clin Ther. 2007;29 Spec No:1284-1292. 18046928

[20] Benson K, Hartz AJ (2000). A comparison of observational studies and randomized, controlled trials. N Engl J Med.

[21] Concato J, Shah N, Horwitz RI (2000). Randomized, controlled trials, observational studies, and the hierarchy of research designs. N Engl J Med.

[22] Meissner Y, Strangfeld A, Costedoat-Chalumeau N, Förger F, Goll D, Molto A, Özdemir R, Wallenius M, Fischer-Betz R (2019). European Network of Pregnancy Registers in Rheumatology (EuNeP)-an overview of procedures and data collection. Arthritis Res Ther.

[23] Radner H, Dixon W, Hyrich K, Askling J (2015). Consistency and utility of data items across European rheumatoid arthritis clinical cohorts and registers. Arthritis Care Res.

[24] Albrecht K, Callhoff J, Edelmann E, Schett G, Schneider M, Zink A (2016). Clinical remission in rheumatoid arthritis. Data from the early arthritis cohort study CAPEA. Z Rheumatol.

