# Supplementary material for: Implementing an automated monitoring process in a digital, longitudinal observational cohort study
Source: Arthritis Res Ther. 2021 Jul 7;23:181. doi: 10.1186/s13075-021-02563-2 (PMC8262053; doi:10.1186/s13075-021-02563-2)
Supplement: Supplementary file 1 — Additional file 1: Detailed description of the monitoring process in RABBIT-SpA. [file 13075_2021_2563_MOESM1_ESM.pdf]

# Supplement

Implementing an automated monitoring process in a digital, longitudinal observational cohort study

# Excerpt of an RABBIT-SpA axspa questionnaire at baseline

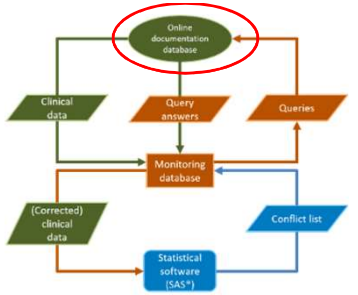


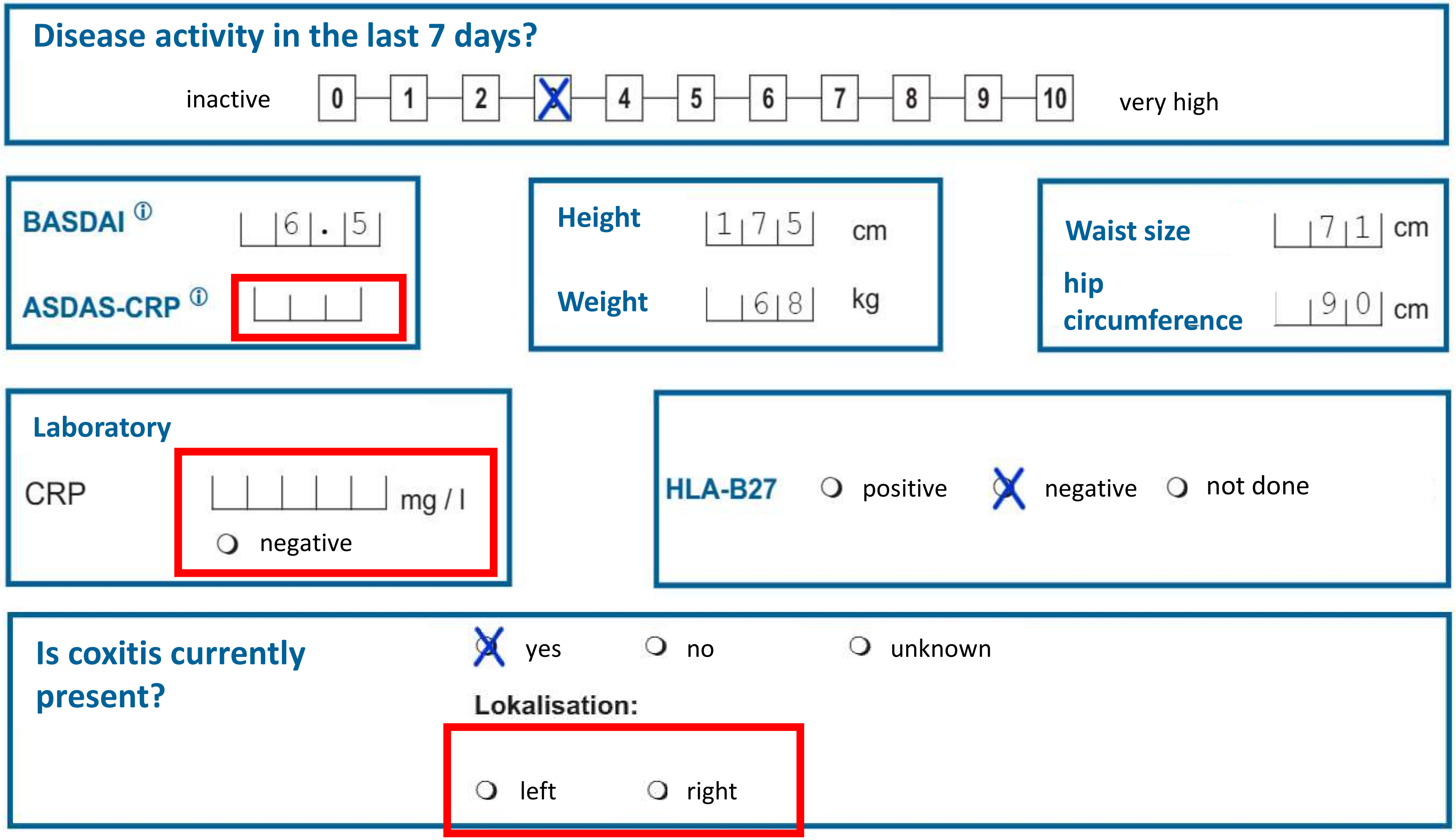

**Disease activity in the last 7 days?**

inactive 0 1 2 X 4 5 6 7 8 9 10 very high

**BASDAI** ⓘ 6 . 5

**ASDAS-CRP** ⓘ

**Height** 1 7 5 cm

**Weight** 6 8 kg

**Waist size** 7 1 cm

**hip circumference** 9 0 cm

**Laboratory**

CRP mg / l

negative

**HLA-B27** positive X negative not done

**Is coxitis currently present?** X yes no unknown

**Lokalisation:**

left right

Example of correct and missing values as displayed in the electronic case report form: disease activity, BASDAI, ASDAS, Height, weight, waist size, hip circumference, CRP, HLA-B27, coxitis.
Plausibility checks are implemented to reduce false entries and subsequent queries.

# Exporting file from documentation system RABBIT-SpA

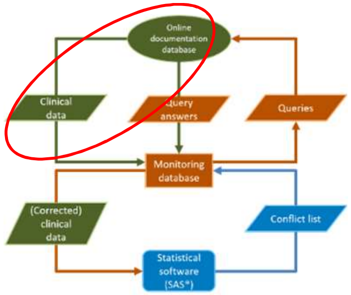


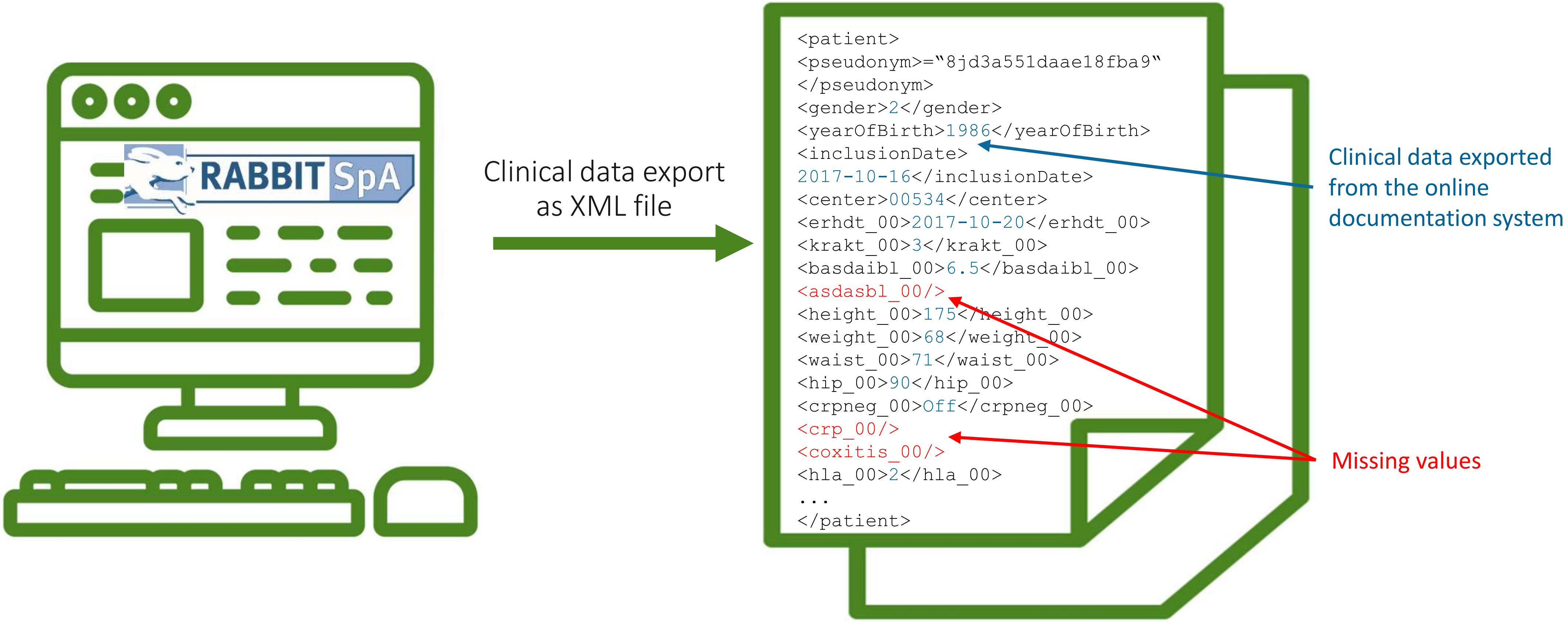


The data manager exports the clinical data from the RABBIT-SpA online documentation system. The variables of all patients are output as an xml file. These variables may contain values or be missing.

# Importing file to monitoring database

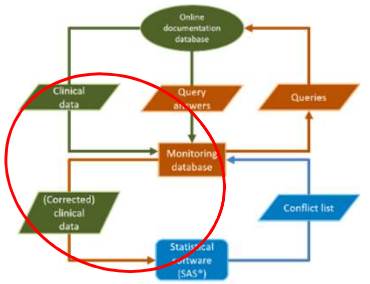



```
<patient>
<pseudonym>="8jd3a551daae18fba9"
</pseudonym>
<gender>2</gender>
<yearOfBirth>1986</yearOfBirth>
<inclusionDate>
2017-10-16</inclusionDate>
<center>00534</center>
<erhdt_00>2017-10-20</erhdt_00>
<krakt_00>3</krakt_00>
<basdaibl_00>6.5</basdaibl_00>
<asdasbl_00/>
<height_00>175</height_00>
<weight_00>68</weight_00>
<waist_00>71</waist_00>
<hip_00>90</hip_00>
<crpneg_00>Off</crpneg_00>
<crp_00/>
<coxitis_00/>
<hla_00>2</hla_00>
...
</patient>
```


Import XML File into RABBIT-SpA monitoring database

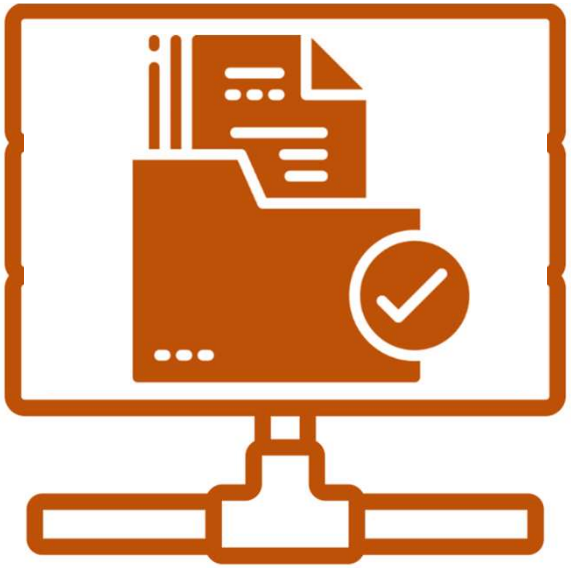

Past query answers and/or corrections outside the monitoring processed within the database

While importing the XML-File to the RABBIT-SpA monitoring database (MDB) the database matches imported information with defined name, type and length of all variables. New variables, which are unknown to the MDB have to be defined. The MDB further provides information about status of patients. After extensive checks on data the MDB creates a SAS file in rectangular structure.

# SAS file created by monitoring database

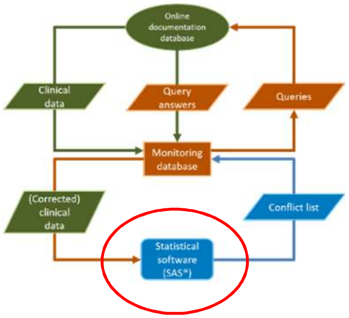


| pseudonym | gender | yearofbirth | inclusiondate | center | erhdt_00 | krakt_00 | basdaibl_00 | asdasbl_00 |
|---|---|---|---|---|---|---|---|---|
| 8jd3a551daae... | male | 1986 | 2017-10-16 | 534 | 2017-10-20 | 3 | 6.5 | . |

| height_00 | weight_00 | waist_00 | hip_00 | crpneg_00 | crp_00 | hla_00 |
|---|---|---|---|---|---|---|
| 175 | 68 | 71 | 90 | . | . | positive |

The SAS dataset is the basis for checking the data. The check is implemented in SAS and one patient per row is displayed with all variables and time points side by side.

# The conflict catalog: Conflict types

**Conflict ID**
Calls the conflict type

**Conflict variables**
for conditions or comparisons

**Conflict type**
All conflicts are categorized into types determining which SAS program is used

**Visit**
Specifies in which follow up visits the conflict can occur. Not all questions occur at each follow up

**Repetition**
Defines the number of maximum repetitions a conflict can be queried

| Conflict ID | Diagnosis | Description | Conflict variables | Check variables | Label | Conflict Type/SAS Macro | conflict block | Visit | Additional variables | Repetition |
|---|---|---|---|---|---|---|---|---|---|---|
| 103 | axSpa /PsA | Year of birth on the CRF does not match the year of birth in the master data. | patient_birthy | birthy | The year of birth on the CRF does not match the year of birth in the master data. Please check "ok" if this is correct and change the value in the master data. | CompareNum | 1 | 0 | ok | 1 |
| 207 | axSpa /PsA | Date of first symptoms before year of birth | birthy | diagy | The initial diagnosis is before the date of birth. Please correct this. If you do not know the start date, please enter "unknown". | CompareNum | 3 | 0 | unk | 1 |
| 803 | axSpA | ASDAS missing | | asdasbl | Please provide the most recent ASDAS available. | MissingNum | 8 | 0 | unk | 1 |
| 1001 | axSpa /PsA | CRP missing | | crp crpneg | Please provide the most recent CRP value. | MissingNum | 10 | 0 1 2 3 4 5 6 7 8 9 10 11 | unk | 1 |
| 1002 | axSpa /PsA | CRP range error | | crp | Der CRP-Wert liegt außerhalb des plausiblen Bereiches. Bitte ändern Sie fehlerhafte Angaben. Ist der Wert richtig so, geben Sie bitte "OK" an. | Range | 10 | 0 1 2 3 4 5 6 7 8 9 10 11 | ok, unk | 1 |
| 1501 | axSpA | Coxitis lokalisation missing | coxlok | cox | Please provide the lokalisation of the coxitis. | MissungBedNum | 24 | 0 | unk | 1 |
| 2012 | axSpA | Dosage increase not plausible | nsa nsa1dedo nsa1deapp | nsa1do nsa1app | No dose increase has occurred (application interval*dose). Please check. | CompareDosage | 60 | 1 2 3 4 5 6 7 8 9 10 11 | unk | 1 |

**Diagnosis**
Diagnosis for which the check is to be performed

**Check variables**
Target variables that are tested

**Label**
Detailed description of the problem and instructions

**Conflict block**
Part of the CRF in which the conflict occurs

**Additional variables**
Can be assigned to any query. Answers can be „ok“, „unknown“ or „not done“

# The conflict catalog: Conflict types

**Conflict types:** All conflicts are assigned a specific conflict type. The most common ones are missing values, dependent missing values, range errors and comparison errors.

**Missing values**: Checks all „check variables“ for simultaneous missings.  One or more variables can be checked. A conflict occurs if all check variables are missing. That means at least one check variable in the column must not be missing. We have divided the questions into blocks because we are presenting excerpts from the CRF and there is only one label for a query block, which can then be worked on together in the group. Eg a CRP value must be given or negative must be checked because this is a mandatory field.

**Dependent missing values:** Checks „check variables“ for simultaneous missing under the condition of the „conflict variables“. A conflict occurs if a „conflict variable“ is missing although another „check variable“ is filled in. Also more complex cases can be checked, eg if a „check variable“ is smaller or greater than a specific value. In this example it is checked whether the lokalisation of the coxitis is filled in under the condition that coxitis was answered with yes.

**Range errors:** Occurs when a value is greater or less than a plausible range of values. The range values are specified in the columns minimum and maximum (which are not shown in this excerpt of the conflict catalog).

**CompareDosage:** Checks if a dosage increase has really occured. If the variable nsa equals 1 (dosage increase) then nsa1do (new dose) x nsa1app (new dose application interval) must be greater than nsa1dedo (old dose) x nsa1deapp (old dose application interval).

**CompareNum:** Checks whether two values are the same. It is checked whether the year of birth (birthy) from the CRF matches the year of birth in the master data (patient_birthy). This is also done for the gender, to make sure that the CRF for the right patient is entered. It can also be checked whether one value is smaller or greater than the other (eg year of first symptoms must be smaller than the year of first diagnosis).

# Creating conflict list in SAS®

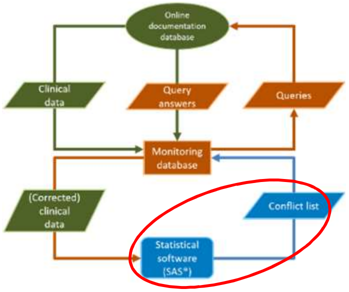


Programming Macros for checks in SAS

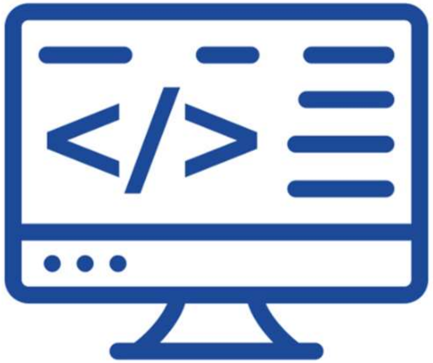

- Each SAS program generates lists per conflict in a uniform format
- The lists are merged at the end to form a total conflict list, which is delivered from SAS to the monitoring database

```
DATA dat91;
        SET dat90;

        ARRAY arpruefvar(*) &pruefvar.;
        n_miss = 0;
        DO i = 1 TO &nopruefvar. ; * Number of check variables ;
                IF arpruefvar(i) EQ . THEN n_miss = n_miss +1;
        END;
        IF n_miss EQ &nopruefvar. THEN fehler = 1;

        KEEP patient_pseudonym &pruefvar. n_miss fehler mzp konfliktmzp zentruma;

        PROC SORT;
        BY patient_pseudonym zentruma KonfliktMZP fehler;
RUN;
* tanspose data so that all variables are among each other ;
PROC TRANSPOSE DATA=dat91 OUT=dat92;
        VAR &pruefvar.;
        BY patient_pseudonym zentruma KonfliktMZP fehler;
RUN;
```

The SAS programs and macros that are used to check the data were specifically programmed for RABBIT-SpA by a statistician. The call is made via the conflict ID in SAS. The program then knows which conflict type must be requested and executed.
For every type of conflict a uniform SAS output is generated and then merged to a total conflict list.

# Processing conflict list in Monitoring Database

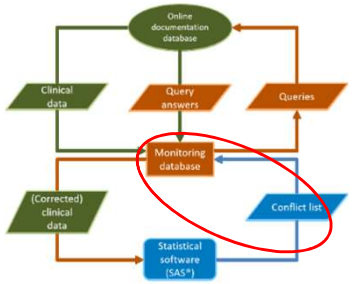


Example for a cumulative conflict list

| Pseudonym | center | conflictID | visit | check_variable | old_value |
|---|---|---|---|---|---|
| 8jd3a551 | 534 | 803 | 0 | asdasbl_ | missing |
| 8jd3a551 | 534 | 1001 | 0 | crp_00 | missing |
| 8jd3a551 | 534 | 1002 | 0 | crpneg_0 | missing |
| 8jd3a551 | 534 | 1501 | 0 | cox_00 | missing |
| 8jd3a551 | 534 | 1502 | 0 | coxli_00 | missing |
| 8jd3a551 | 534 | 1503 | 0 | coxre_00 | missing |

Import conflict list into monitoring database

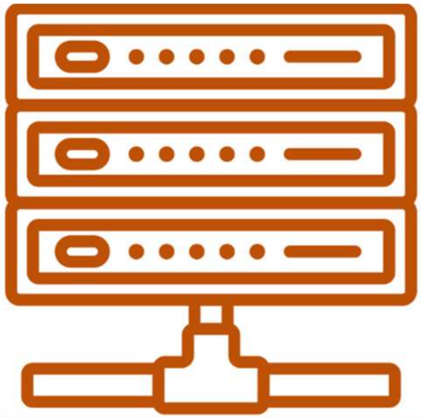

The imported conflict list is processed in the monitoring database. After the process is triggered by the data manager, the monitoring database decides whether a conflict is turned into a query or not. A query is created if the number of repetitions has not yet been reached, none of the additional variables have been checked in a previous query and it is therefore answered or queries have not yet been returned and are still pending. The result is an XML file which is uploaded to the online documentation system RABBIT-SpA.

# Processing conflict list in Monitoring Database

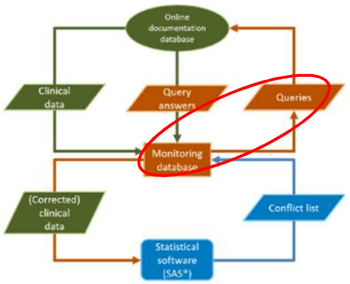


| Conflict ID | Diagnosis | Description | Conflict variables | Check variables | Label | Conflict Type/SAS Macro | conflict block | Visit | Additional variables | Repetition |
|---|---|---|---|---|---|---|---|---|---|---|
| 803 | axSpA | ASDAS missing | asdas | asdasbl | Please provide the most recent ASDAS available. | MissingBedNum | 8 | 0 | unk | 1 |
| 1001 | axSpA | CRP missing | | crp crpneg | Please provide the most recent CRP value. | MissingNum | 10 | 0 1 2 3 4 5 6 7 8 9 10 11 | unk | 1 |


```
</Query_ID><Query_ID
Query_ID="9648">
<ConflictID="803">
<Pseudonym>8jd3a551da
ae18fba9</Pseudonym>
<Visit>0</Visit>
<Label>Please provide the
most recent CRP
value.</Label>
<ok/>
<not applicable/>
<unknown/>x</unknown>
</Query_ID><Query_ID
Query_ID="9648">
<ConflictID="1001">
<Pseudonym> 8jd3a551da
ae18fba9</Pseudonym>
<Visit>0</Visit>
<Label>Please provide
The most recent ASDAS
available.</Label>
<ok/>
<not applicable/>
<unknown>x</unknown>
```


The monitoring database uses several variables from the conflict catalog to create queries. The conflict ID is used by the monitoring database to assign a label and additional variables to a query and the number of repetitions to decide whether to call a query or not.

# Presentation of a query within the documentation system

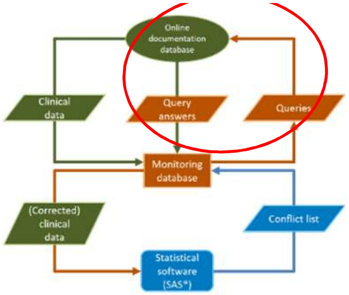


Queries regarding visit T 0 from date

Name

Laboratory

CRP |_|_|_|_|_| mg / l

❍ negative

Please provide the most recent CRP value.

❐ unknown

BASDAI ⓘ |_|6|.|5|

ASDAS-CRP ⓘ |_|_|_|

Please provide the most recent ASDAS.

❐ unknown

To make answering the queries as easy and comfortable as possible for participating physicians we are displaying an excerpt of the original eCRF, instead of, eg, sending an excel list. This gives the physician the chance to recheck the queried values and relevant associated values. This makes it particularly easy to correct or change either the queried value or, if necessary, an associated value within the shown query block.

# Corrections structured in XML-File

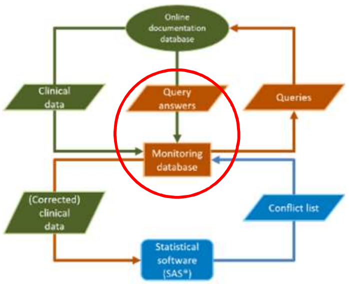


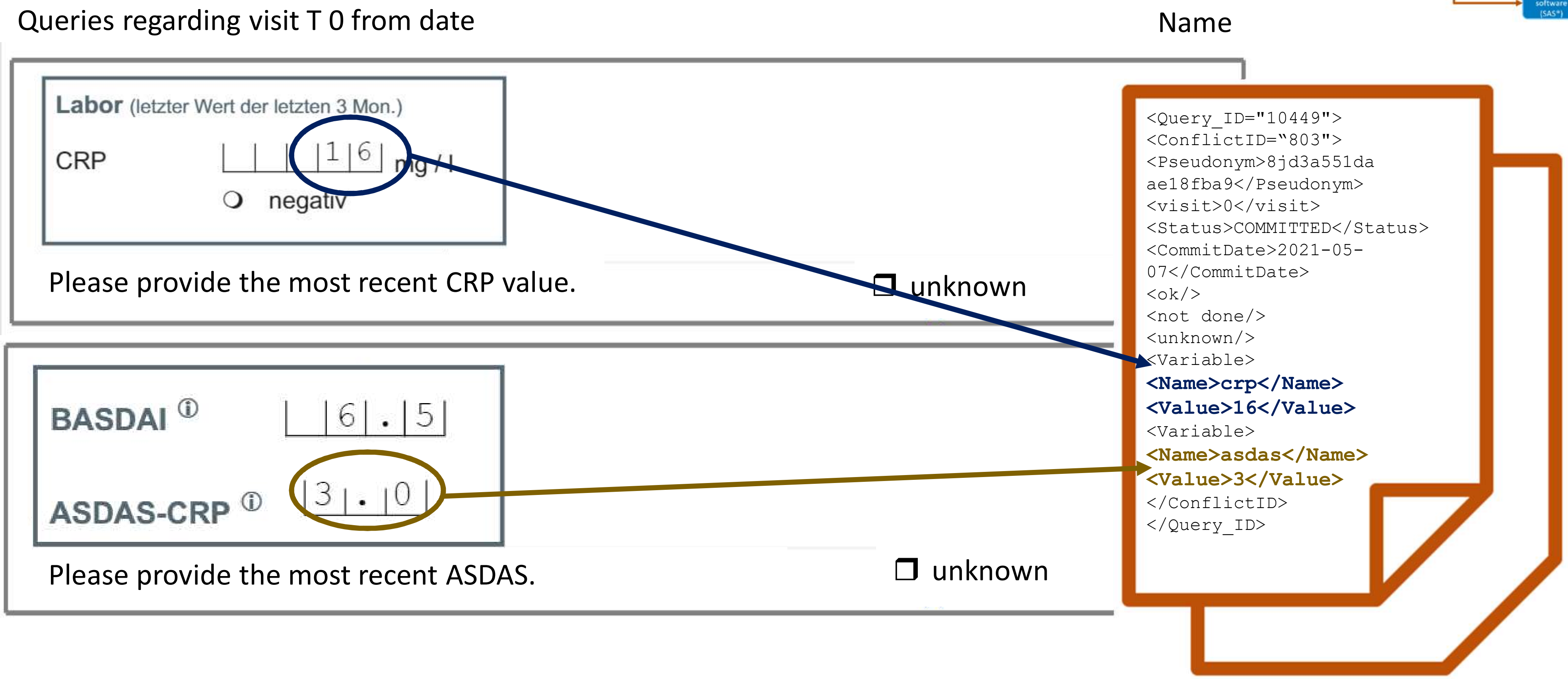


After the physician has answered the queries the data manger downloads the xml file with query corrections. This file is imported into the database, which updates the clinical data with corrections and creates a corrected SAS file.
The new values overwrite the old or missing values in the clinical data. All changes made in the data are saved, to make changes traceable.
